# Is Vancomycin More Effective than Taurolidine? Comparative Analysis of Their Preventive Effect against Spinal Infection in 1000 Patients with Spinal Fusion

**DOI:** 10.3390/antibiotics11101388

**Published:** 2022-10-11

**Authors:** Dong-Chan Eun, Kyung-Soo Suk, Hak-Sun Kim, Ji-Won Kwon, Seong-Hwan Moon, Yong-Ho Lee, Byung-Ho Lee

**Affiliations:** Department of Orthopedic Surgery, College of Medicine, Yonsei University, Seoul 120-752, Korea

**Keywords:** taurolidine irrigation, vancomycin powder application, surgical site infection

## Abstract

This study aimed to examine the effect of taurolidine irrigation on preventing surgical site infection by comparing the spinal infection rate after spinal fusion surgery using vancomycin powder application and taurolidine irrigation. Of 1081 participants, 369 underwent taurolidine irrigation, 221 underwent vancomycin powder application, and 491 were controls. Of the 20 surgical site infections (1.85%), 14 occurred in the control group (2.85%), 5 in the vancomycin group (2.26%), and 1 (0.27%) in the taurolidine group. Among the various variables, age at the time of surgery, smoking, surgical site, and hemovac removal time were significant in the univariate logistic regression. The final result was derived after variable selection using the stepwise method. In the univariate model, the odds ratios were 0.09 and 0.79 in each of the vancomycin and taurolidine groups compared to that of the control group. In the multivariate model, the odds ratios were 0.09 and 0.83 in each of the vancomycin and taurolidine groups compared to that of the control group. The preventive effect of vancomycin powder application was not statistically significant. However, the vancomycin group showed a less effective tendency than the taurolidine group. Taurolidine irrigation may be a good substitute for the vancomycin powder application.

## 1. Introduction

Surgical site infection is a common healthcare-associated infection and complicates up to 10–20% of operations with considerable strain on healthcare resources [1]. Surgical site infection is the most common cause of nosocomial infections and is defined as the failure of a patient’s immune response to resident bacteria in the hospital. The incidence of surgical site infection after spinal surgery has been reported to have a large deviation of 0–15% [2,3,4,5,6].

Spinal surgery is challenging and tends to be invasive; therefore, it has a higher risk of infection than other orthopedic surgeries. Advances in surgical instruments, surgical techniques, and anesthesia have made spinal surgery increasingly available for difficult and complex spinal diseases. Unfortunately, diagnosing and treating more complex spinal diseases results in a higher infection rate after surgery. The infection rate after a simple discectomy or posterior laminectomy is approximately 1%, while that after spinal fusion is 2–5%. Adding posterior instrumentation to spinal fusion increases the risk of infection by 2.4–8.5%. The most frequently infected spinal fusion is anterior-posterior combined spinal fusion [7,8,9].

Locally applied antibiotics date back to the mid-1800s when Joseph Lister began using carbolic acid as an antibiotic on surgical wounds. The modern use of local antibiotics in orthopedics began with the use of polymethylmethacrylate (PMMA) cement impregnated with antibiotics to treat infected joint prostheses. The use of locally applied antibiotics allows delivery of high concentrations of antibiotics without significant risk of systemic toxicity and has been shown to be effective against biofilms. In addition, vancomycin powder application or aqueous gentamycin irrigation is used as an antibiotic without a carrier and gel, sponge, or calcium sulfate-containing antibiotics are also used as local antibiotics [10,11].

Since vancomycin’s first application in 2011, it has been used locally in many medical institutions to prevent surgical site infections. However, the effects of local vancomycin administration were not constant. Moreover, the pharmacokinetics of vancomycin powder application remains unclear [12,13,14].

Taurolidine is an antimicrobial agent frequently used in experimental and clinical studies to prevent intra-abdominal adhesions and sepsis. Initially, it was used to obtain anti-inflammatory and antibacterial effects in surgery and wound infection treatment and to prevent infection caused by central vein catheters. However, there are few studies on the effect of taurolidine in orthopedic surgery. In particular, research is yet to be conducted to determine whether taurolidine can reduce the risk of infection after spinal fusion [15,16,17].

Therefore, this study aimed to compare the effect of vancomycin powder application to that of taurolidine irrigation in preventing spinal infection at the surgical site after spinal fusion.

## 2. Materials and Methods

### 2.1. Patient Selection

Patients who underwent anterior or posterior spinal fusion treatment for degenerative diseases of the cervical, thoracic, and lumbar spine at the Sinchon Severance Hospital and Gangnam Severance Hospital between February 2018 and February 2022 were included in this study. Patients with infectious diseases, such as infectious spondylitis, intervertebral discitis, abscesses in the spine, primary or metastatic cancer, open fractures or wounds, and a history of infectious diseases in other body parts and those taking immunosuppressants or antibiotics before hospitalization were excluded from this study. This retrospective study was approved by the clinical trial review board of the hospital. Of 1081 participants, 369 underwent taurolidine irrigation, 221 underwent vancomycin powder application, and 491 were controls. The control group did not receive either vancomycin powder or taurolidine irrigation.

### 2.2. Preparation and Process of Surgery

In all patients, 1 g of a prophylactic antibiotic that was administered after surgery was administered intravenously within an hour of surgery. Furthermore, most patients were administered 1 g of cefazolin as a preventive antibiotic. The operating room environment, including temperature, humidity, and ventilation, or the surgeon, assistant, and nurse was the same for all participants, and all participants wore disposable surgical clothes. After anesthesia, one assistant wiped the surgical site with 7.5% betadine solution and then disinfected the surgical site with 10% betadine solution and alcohol again. The exposed area was sealed using Surgidrap. After the main procedure was completed, sufficient saline was irrigated in all cases, and taurolidine or vancomycin powder was applied before the drainage tube was inserted. In the case of taurolidine (Taurolidine injection 2%, Samjin, Seoul, Korea), one bottle of taurolidine was heated to 37 °C, 750 mL of saline was mixed with 250 mL of taurolidine, and a total of 1 L was irrigated with bones, muscles, fascia, subcutaneous fat, and dermis using an irrigation spoid at the surgical site. In the case of vancomycin (vancomycin HCl injection 1 g, inno.N, Seoul, Korea), vancomycin powder was finely crushed, and approximately 2/3 was applied to the bones and muscles. The remaining powder was applied to the subcutaneous fat and dermis. The amount of vancomycin applied varied between 0.5–2 g depending on the wound size and surgical type; however, most patients were administered 1 g. After surgery, the same preventive antibiotics used preoperatively were administered intravenously until the first postoperative day.

### 2.3. Patient Evaluation

Participants’ characteristics, including sex, age, height, weight, body mass index, history of smoking, high blood pressure, diabetes, kidney disease, respiratory disease, preoperative white blood cell (WBC) and neutrophil counts, history of spinal surgery and spinal infection, postoperative blood sugar level, instrumented fusion level, bone graft type, bleeding during surgery, blood transfusion, time of hemovac removal, and volume of hemovac drainage were collected. Since posterior implantation was performed in all participants, it was determined by following up for 90 days after surgery according to the WHO standards [18]. In addition, surgical site infections were classified as superficial infections at the incision site and deep infections following the standard surveillance criteria of the hospital infection supervisory authority (NNIS System) under the U.S. Centers for Disease Control and Prevention (CDC) [19]. Clinical visual examination of the surgical site, pain and tenderness, edema, redness, heating sense, and body temperature changes were observed, and WBC count, red blood cell (RBC) sedimentation rate (ESR), and C-reactive protein (CRP) were measured using hematological infection markers. Magnetic resonance imaging (MRI) was performed when surgical site infection was suspected, and surgical incision and drainage were performed in patients with correlated clinical and imaging findings after MRI. A bacterial identification test was performed using a bacterial culture medium for drainage, and tissue culture (for example, bone or fascia) was performed, if necessary.

### 2.4. Statistical Analysis

A statistical analysis of the Kruskal–Wallis test was conducted for numerical variables between groups, and the chi-square test was conducted for categorical variables. Univariate logistic regression was performed to determine the factors affecting the infection status. After univariate logistic regression was performed for each independent variable, multivariate regression analysis was performed. Variable selection was performed using the stepwise method for significant variables in the univariate logistic regression, and the final model result was indicated. Statistical analysis was performed using SAS version 9.4 (SAS Institute, Cary, NC, USA). Statistical significance was set at a *p*-value ≤ 0.05.

## 3. Results

### 3.1. Patient Demographics

Patients’ characteristics and the descriptive statistics between the patient groups are summarized in Table 1. A total of 518 male and 563 female patients were included, with a median age of 65 years (range 20–93 years). For the body mass index of the patients, 723 were under 25 (66.88%), 283 were between 25 and 30 (26.18%), and 75 were over 30 (6.94%). The smoking rate was 7.68% (83 patients). The rates of high blood pressure, diabetes, kidney disease, and respiratory disease were 46.07% (498 patients), 22.02% (238 patients), 0.83% (nine patients), and 0.46% (five patients), respectively. History of spinal surgery and spinal infection was observed in 46 (4.26%) and three (0.28%) patients, respectively. Surgical sites were 343 cervical vertebrae (31.73%), 33 thoracic vertebrae (3.05%), 52 thoracolumbar vertebrae (4.81%), 550 lumbar vertebrae (50.88%), and 103 lumbosacral vertebrae (9.53%). The median instrumented fusion level was 2 (range 1–7). The median preoperative WBC count and absolute neutrophil count (ANC) were 6.89 × 10^3^/μL (range 2.29–13.62 × 10^3^/μL) and 4416.40 N/μL (range 1178.52–8748.29 N/μL), respectively. The median amount of bleeding during surgery was 350 cc (range 10–2500 cc), and 200 patients (18.50%) received blood transfusions during surgery. Regarding bone graft type during surgery, 952 patients (88.07%) received autobone grafts, and 129 (11.93%) received autobone and allobone grafts. The median time of hemovac removal after surgery was 3 postoperative days (range 1–11 days), and the median cumulative amount of hemovac was 380.40 cc (range 10–2697.80 cc). After surgery, the median serum glucose and albumin levels (g/dL) were 128.5 (range 53–304) and 3.5 (range 1.70–5.00), respectively. Comparing patient information among the three groups showed no statistical difference in all variables except surgical site and preoperative ANC.

### 3.2. Surgical Site Infection Evaluation

Of the 20 infected patients, infection occurred in the lumbar vertebrae (60%) in 12 cases, the lumbosacral vertebrae (20%) in four cases, and the thoracolumbar vertebrae (20%) in four cases.

Fourteen cases (70%) of superficial infections and six cases (30%) of deep infections occurred. Regarding superficial infections, 10 cases, three cases, and one case were observed in the control, vancomycin, and taurolidine groups, respectively. They were treated using secondary sutures after debridement and sufficient irrigation in the ward or operating room, depending on the wound size. The mean postoperative infection date was 14 ± 5 days.

Regarding deep infections, four cases, two cases, and no cases were observed in the control, vancomycin, and taurolidine groups, respectively. They were treated using secondary sutures after debridement and sufficient irrigation in the operating room. The mean postoperative infection date was 16 ± 6 days.

Microbiological analysis performed during surgery in all patients revealed that 65% (13/20) of infections were caused by *Staphylococcus aureus* (nine cases vs. four cases vs. zero cases in control, vancomycin, and taurolidine groups, respectively). The remaining (35%) were caused by gram-negative bacteria in five cases of *Escherichia coli* isolates (three cases vs. one case vs. one case in the control, vancomycin, and taurolidine groups, respectively), one case of *Klebsiella pneumoniae*, and one case of *Pseudomonas aeruginosa* (Table 2 and Table 3).

### 3.3. Risk Factors for Surgical Site Infections

Univariate logistic regression was performed to determine the factors affecting the infection status. After univariate logistic regression was performed for each independent variable, multivariate regression analysis was performed. Among the various variables, age at the time of surgery, smoking, surgical site, and hemovac removal time were significant in the univariate logistic regression. The final result was derived after variable selection using the stepwise method. In the univariate model, the *p*-value of taurolidine was 0.022; therefore, it can be said that the use of taurolidine affects infection. The risk of infection in the taurolidine group was 0.09 times lower than that in the control group. In the multivariate model, the *p*-value of taurolidine was 0.02; therefore, it can be said that taurolidine affects infection while correcting the correction variables (age at the time of surgery, smoking, and hemovac removal). The risk of infection in the taurolidine group was 0.09 times lower than that in the control group. In the vancomycin group, the *p*-value was less than 0.05, and there was no significant difference; however, in the univariate model, the risk of infection in the vancomycin group was 0.79 times higher than that in the control group. In the multivariate model, the risk of infection in the vancomycin group was 0.83 times higher than in the control group (Table 4). 

## 4. Discussion

Vancomycin is a glycopeptide. Initially, it was used to treat cases of inflammation caused by *Staphylococcus aureus* in which the patient had a penicillin allergy or could not be treated with penicillin due to bacterial resistance [20]. Vancomycin attacks the bacterial cell wall and causes various defects, such as changes in the permeability of bacterial cell membranes and selective inhibition of the formation of several RNAs. When vancomycin is applied locally, high concentrations of antibiotics accumulate at the surgical site while maintaining the drug concentration in the blood flow, killing gram-positive bacteria at the surgical site and not harming the internal organ. In the first report by Sweet et al., where vancomycin was applied as a means of preventing infection at the surgical site in spinal surgery, 1 g vancomycin was applied to the bone graft after surgery, and 1 g vancomycin was applied to the incision area (0.2%), which was significantly lower than that in the control group (2.6%) [21]. Blood flow disturbances usually occur after spinal injury or surgery, making it difficult to deliver antibiotics to the area through the veins. Local administration of vancomycin helps form sufficient antibiotic concentrations in the damaged area while avoiding the side effects of high drug concentrations in the circulatory system. Furthermore, most studies have confirmed that vancomycin can significantly reduce the incidence of surgical site infections. In this study, while there was no significant difference between the vancomycin and control groups in the univariate logistic regression analysis, the odds ratio was 0.789; therefore, the possibility of infection tends to decrease when vancomycin is used. However, there is no consensus on the effective amount of vancomycin to be applied. Various studies used vancomycin application amounts ranging from 0.5–6 g, and doses of 1–2 g were used in most studies. The area in which vancomycin should be administered is controversial. There is disagreement as to whether vancomycin should be applied to the deep or surface fascia, directly to bone grafts, or to the host bones. In addition, intravenous administration of vancomycin can cause thrombotic venous inflammation, eosinophilia, Redman syndrome, ototoxicity, and nephrotoxicity [22]. Mariappan reported the occurrence of allergic reactions and circulatory collapse in female patients administered 1 g of vancomycin powder after breast cancer metastasis, and Youssef reported epidural seroma 6 weeks after local application of 2 g vancomycin to female patients undergoing lumbar surgery [23,24].

In addition to safety, drug resistance is an important problem associated with antibiotic applications. Because vancomycin is one of the most effective treatments for surgical infections, whether local application of vancomycin can lead to vancomycin-resistant bacterial infection is one of the main concerns in the preventive application of this antibiotic.

Taurolidine [bis (1,1-dioxo-perhydro-1,2,4-thiadiazinyl-4) methane] was introduced in the 1970s and was originally used as an antibiotic to prevent bacterial infections in the abdominal cavity of patients with peritonitis [25]. In addition to proper surgical treatment and postoperative management, the initial local administration of taurolidine plays an important role in preventing postoperative sepsis complications and is known to reduce postoperative mortality [26]. In addition, the usefulness of taurolidine as an anticancer drug has been extensively investigated in recent years [27]. Taurolidine contains a wide range of non–antibiotic antibacterial agents chemically derived from taurine, an amino sulfonic acid. The antibacterial effect of taurolidine is due to the action of a biologically active methylol taurinamide component that reacts with the cell wall component of bacteria through methylene iminium ions [28].

Taurolidine has a unique spectrum of antibacterial activity against gram-positive and gram-negative bacteria and fungi [29,30]. Taurolidine exhibits bactericidal effects on many bacterial species, including *Staphylococcus aureus*, *Staphylococcus epidermidis*, *Bacteroides fragilis*, and group C streptococci [31]. Taurolidine has long been used in treating abdominal and lung infections and in dentistry [32]. The most common bacteria that cause infections after spinal surgery are *Staphylococcus aureus*, *Staphylococcus epidermidis*, and *Streptococcus* species. Therefore, we assumed that taurolidine could contribute to reducing the risk of infection after spinal fusion surgery.

This study showed the effectiveness of taurolidine irrigation in over 1000 patients who underwent spinal fusion. Of the 369 patients who received taurolidine, only one patient was infected. Compared to the patient group treated with vancomycin powder, infection occurred with a much smaller probability in both patient groups without vancomycin. In addition, comparing the control and taurolidine groups, the odds ratio was 0.09 in the univariate regression analysis and 0.09 in the multivariate regression analysis, showing very effective infection prevention by taurolidine. In this study, no side effects of taurolidine were found.

The risk factors for postoperative infection can be largely divided into patient and surgical factors, including revision surgery, infectivity in other areas, diabetes, smoking, obesity, malnutrition, poor immune function, and long–term hospitalization before surgery [1,33,34,35]. In this study, smoking showed a statistically significant difference. Fang et al. reported that diabetes, age >60 years, smoking, drinking, history of infection, and obesity were risk factors in the analysis of 48 infected patients (4.4%) in 1095 surgeries [7]. Simpson et al. reported in a retrospective study of patients under decompression that infection occurred in 24% of 62 diabetic patients, and no infection occurred in the control group [36]. In this study, diabetes morbidity did not cause statistical differences because there were few cases of morbidity, and the blood sugar concentration of diabetic patients was relatively well controlled to 83–230 mg/dL before surgery. Malnutrition or decreased immune function are also factors that increase postoperative infection. Stambough et al. reported an albumin concentration of 3.5 g/dL or less or a total number of lymphocytes of 2000/μL or less as risk factors for postoperative infection [37]. The basal albumin levels in this study did not show any statistical differences among the groups, indicating a median of 3.5 g/dL in all patients. The median ANC exceeded 4000 N/μL in all three groups.

Surgical factors included the type of surgery, presence of instrumentation, the extent of dissection, time of surgery, and amount of bleeding. Wimmer et al. reported no infections in 276 anterior approaches; however, 3.8% of infections occurred in posterior approaches [38]. In this study, consistent with the above study, infection occurred in all cases that used the posterior approach. The rate of infection after partial discectomy has been reported to be 0.4–2.9%; however, infection was reported to be 2% when fusion is performed and 4–6% when posterior implantation and fusion are performed. It is thought that important surgical factors are correlated with tissue exposure. In addition, postoperative infections have been reported to increase even in cases of bleeding of more than 1000 cc or surgery of more than 3 h. In this study, loss of bleeding, packed RBC transfusion, hemovac removal time, and the accumulated volume of hemovac were measured as factors associated with bleeding. Among them, the odds ratio of packed RBC transfusion was 3.02, and that of the accumulated volume of hemovac was 1.41, showing a statistically significant difference. 

Preventive systemic antibiotics should also be considered. In this study, cefazolin was administered to patients before and after surgery at the same dose and at the same time. Preventive antibiotics are not administered as a concept to sterilize tissue, but as a concept of helping to keep the load of microorganisms infected during surgery below the host defense level and are not preventing infection through postoperative incision wounds [39]. Therefore, the effectiveness of different doses and continuous administration of types of preventive antibiotics is still controversial. Kanayma reported that there was no difference in the incidence of infection in the past between the results of preventive antibiotics for 5–7 days after surgery and the results of administration only on the day of surgery [40].

This study had some limitations. First, it was impossible to present an appropriate use of preventive taurolidine except for intermittent irrigation at the surgical site with a 0.5% taurolidine injection heated to 37 °C. Second, because this was a retrospective study, the same number of patients could not be matched for each surgical site. Finally, it was impossible to include all the diverse risk factors for spinal infection.

## 5. Conclusions

Surgical site infections after spinal fusion can lead to serious morbidity and mortality. This study showed that taurolidine irrigation could effectively prevent infection at the surgical site after spinal fusion. It may be an excellent substitute to compensate for the complications and problems associated with vancomycin powder application.

## Figures and Tables

**Table 1:** Baseline characteristics of the study groups.

	Median (Min–Max) or N(%)				*p*-Value
	Total (*n* = 1081)	Group			
		Control (*n* = 491)	Vancomycin (*n* = 221)	Taurolidine (*n* = 369)	
Age	65.00 (20.00–93.00)	64.00 (40.00–87.00)	65.00 (22.00–93.00)	65.00 (20.00–85.00)	0.294
Sex					0.910
Male	518 (47.92)	234 (47.66)	104 (47.06)	180 (48.78)	
Female	563 (52.08)	257 (52.34)	117 (52.94)	189 (51.22)	
Body mass index (m/kg^2^)					0.468
<25	723 (66.88)	321 (65.38)	156 (70.59)	246 (66.67)	
25 ≤ < 30	283 (26.18)	139 (28.31)	51 (23.08)	93 (25.20)	
30≤	75 (6.94)	31 (6.31)	14 (6.33)	30 (8.13)	
Infection					0.019 **
NO	1061 (98.15)	477 (97.15)	216 (97.74)	368 (99.73)	
YES	20 (1.85)	14 (2.85)	5 (2.26)	1 (0.27)	
Smoking					0.943
NO	998 (92.32)	452 (92.06)	204 (92.31)	342 (92.68)	
YES	83 (7.68)	39 (7.94)	17 (7.69)	27 (7.32)	
Hypertension					0.104
NO	583 (53.93)	282 (57.43)	111 (50.23)	190 (51.49)	
YES	498 (46.07)	209 (42.57)	110 (49.77)	179 (48.51)	
Diabetes mellitus					0.371
NO	843 (77.98)	387 (78.82)	177 (80.09)	279 (75.61)	
YES	238 (22.02)	104 (21.18)	44 (19.91)	90 (24.39)	
Pulmonary disease					0.910
NO	1072 (99.17)	486 (98.98)	220 (99.55)	366 (99.19)	
YES	9 (0.83)	5 (1.02)	1 (0.45)	3 (0.81)	
Renal disease					1.000
NO	1076 (99.54)	489 (99.59)	220 (99.55)	367 (99.46)	
YES	5 (0.46)	2 (0.41)	1 (0.45)	2 (0.54)	
History of spinal surgeries					0.612
NO	1035 (95.74)	471 (95.93)	209 (94.57)	355 (96.21)	
YES	46 (4.26)	20 (4.07)	12 (5.43)	14 (3.79)	
History of spinal infection					0.789
NO	1078 (99.72)	490 (99.80)	220 (99.55)	368 (99.73)	
YES	3 (0.28)	1 (0.20)	1 (0.45)	1 (0.27)	
Surgical site					0.017 **
Cervical	343 (31.73)	142 (28.92)	71 (32.13)	130 (35.23)	
Thoracic	33 (3.05)	12 (2.44)	2 (0.90)	19 (5.15)	
Thoraco-lumbar	52 (4.81)	30 (6.11)	10 (4.52)	12 (3.25)	
Lumbar	550 (50.88)	256 (52.14)	121 (54.75)	173 (46.88)	
Lumbo sacral	103 (9.53)	51 (10.39)	17 (7.69)	35 (9.49)	
Bone graft					0.409
Autobone	952 (88.07)	434 (88.39)	199 (90.05)	319 (86.45)	
Autobone + allobone	129 (11.93)	57 (11.61)	22 (9.95)	50 (13.55)	
Transfusion (packed RBC)					0.089
No	881 (81.50)	392 (79.84)	175 (79.19)	314 (85.09)	
Yes	200 (18.50)	99 (20.16)	46 (20.81)	55 (14.91)	
Instrumented fusion level	2.00 (1.00–7.00)	2.00 (1.00–5.00)	2.00 (1.00–6.00)	2.00 (1.00–7.00)	0.381
Preoperative WBC count (10^3^/μL)	6.89 (2.29–13.62)	6.92 (2.29–13.62)	6.69 (2.90–11.98)	6.95 (3.48–10.99)	0.989
Preoperative absolute neutrophil count (N/μL)	4416.40 (1178.52–8748.29)	4653.39 (1178.52–8748.29)	4619.83 (1399.65–8415.68)	4031.04 (1831.38–8337.85)	<0.001 **
Estimated blood loss (cc)	350.00 (10.00–2500.00)	300.00 (10.00–1890.00)	400.00 (10.00–1900.00)	350.00 (10.00–2500.00)	0.081
Postoperative Hemovac removal (POD)	3.00 (1.00–11.00)	3.00 (1.00–9.00)	3.00 (1.00–8.00)	3.00 (1.00–11.00)	0.271
Accumulated volume ofHemovac (cc)	380.40 (10.00–2697.80)	370.80 (10.00–2697.80)	415.80 (20.00–2118.00)	321.00 (10.00–2810.00)	0.115
Postoperative serum glucose	128.50 (53.00–304.00)	128.00 (53.00–291.00)	130.00 (83.00–254.00)	128.00 (78.00–304.00)	0.754
Postoperative serum albumin (g/dL)	3.50 (1.70–5.00)	3.50 (2.00–4.70)	3.60 (1.80–4.80)	3.50 (1.70–5.00)	0.201

** *p*–value ≤ 0.05.

**Table 2:** Microbiological analysis of the identified surgical site infections.

Isolated Microbes	Superficial Infections (*n*)	Deep Infections (*n*)
*Staphylococcus epidermidis*	3	1
*Staphylococcus aureus*	2	1
Methicillin-Resistant Coagulase Negative *Staphylococcus*	3	1
Methicillin-Resistant *Staphylococcus aureus*	1	1
*Escherichia coli*	3	2
*Klebsiella pneumonia*	1	0
*Pseudomonas aeruginosa*	1	0
Total number	14	6

**Table 3:** Isolated microbes in infections in each group.

Isolated Microbes *	Total (*n* = 20)	Control (*n* = 14)		Vancomycin (*n* = 5)		Taurolidine (*n* = 1)	
		Suferficial Infections (*n*)	Deep Infections (*n*)	Suferficial Infections (*n*)	Deep Infections (*n*)	Suferficial Infections (*n*)	Deep Infections (*n*)
*S. epidermidis*	4	2 (50%)		1 (25%)	1 (25%)		
*S. aureus*	3	1 (33%)	1 (33%)	1 (33%)			
MRCNS	4	2 (50%)	1 (75%)	1 (25%)			
MRSA	2	1 (50%)	1 (50%)				
*E. coli*	5	2 (40%)	1 (20%)		1 (20%)	1 (20%)	
*K. pneumoniae*	1	1 (100%)					
*P. aeruginosa*	1	1 (100%)					
Total number	20	10 (50%)	4 (20%)	3 (15%)	2 (10%)	1 (5%)	0 (0%)

* Samples for culture were taken from a swab as deep as possible in the ward or operating room based on the presence of signs or symptoms of infection. In some cases, tissue cultures (for example, bone or fascia) were obtained in the operating room under general anesthesia in case of revision or debridement.

**Table 4:** Logistic regression of performing to determine what factors affect surgical site infections.

	Univariable		Multivariable	
	OR (95% CI)	*p*-Value	OR (95% CI)	*p*-Value
Age	1.07 (1.02–1.12)	0.004 **	1.07 (1.02–1.12)	0.007 **
Sex				
Male	Ref			
Female	1.39 (0.563–3.42)	0.476		
Body mass index (m/kg^2^)				
<25	Ref			
25 ≤ < 30	0.68 (0.22–2.06)	0.491		
30≤	0.64 (0.08–4.90)	0.666		
Infection				
Control	Ref		Ref	
Vancomycin	0.79 (0.21–2.22)	0.653	0.83 (0.27–2.55)	0.739
Taurolidine	0.09 (0.01–0.71)	0.022 **	0.09 (0.01–0.68)	0.020 **
Smoking				
NO	Ref		Ref	
YES	6.98 (2.71–18.01)	<0.001 **	11.23 (3.92–32.20)	<0.001 **
Hypertension				
NO	Ref			
YES	0.63 (0.25–1.58)	0.320		
Diabetes mellitus				
NO	Ref			
YES	2.41 (0.97–5.96)	0.057		
Pulmonary disease				
NO	Ref			
YES	2.70 (0.13–55.88)	0.520		
Renal disease				
NO	Ref			
YES	4.68 (0.19–115.10)	0.345		
Past history of spinal surgeries				
NO	Ref			
YES	0.53 (0.03–9.21)	0.665		
Past history of spinal infection				
NO	Ref			
YES	7.37 (0.23–232.26)	0.256		
Surgical site				
Cervical	Ref			
Thoracic	1.45 (0.07–29.80)	0.809		
Thoraco-lumbar	6.88 (1.50–31.44)	0.013 **		
Lumbar	2.26 (0.68–7.46)	0.181		
Lumboscral	2.40 (0.46–12.40)	0.297		
Bone graft				
Autobone	Ref			
Autobone + allobone	0.18 (0.010–2.952)	0.227		
Transfusion (packed RBC)				
No	Ref			
Yes	3.02 (1.22–7.48)	0.017 **		
Instrumented fusion level	1.08 (0.86–1.36)	0.492		
Preoperative WBC count (10^3^/μL)	0.86 (0.67–1.09)	0.205		
Preoperative absolute neutrophil count (N/μL)	1.00 (1.00–1.000)	0.085		
Estimated blood loss during surgery (cc)	1.00 (1.00–1.00)	0.090		
Postoperative Hemovac removal (POD)	1.41 (1.13–1.76)	0.002 **	1.38 (1.08–1.77)	0.010 **
Accumulated volume of Hemovac (cc)	1.00 (1.00–1.00)	0.054		
Postoperative serum glucose	1.00 (0.99–1.02)	0.526		
Postoperative serum albumin (g/dL)	0.56 (0.23–1.36)	0.201		

** *p*–value ≤ 0.05.

## Data Availability

Not applicable.
